# Comparison of 5-year cup and stem migration between ceramic-on-ceramic and ceramic-on-polyethylene bearing in press-fit total hip arthroplasty: a randomised controlled trial using radiostereometric analysis

**DOI:** 10.1177/11207000241265653

**Published:** 2024-08-19

**Authors:** Amanda D Klaassen, Justin van Loon, Nienke W Willigenburg, Lennard A Koster, Bart L Kaptein, Victor P M van der Hulst, Daniel Haverkamp, Dirk Jan F Moojen, Rudolf W Poolman

**Affiliations:** 1 Department of Orthopaedic Surgery, OLVG, Amsterdam, the Netherlands; 2 Department of Orthopaedic Surgery, LUMC, Leiden, the Netherlands; 3 Xpert Clinics Orthopedie Amsterdam, the Netherlands; 4 Department of Orthopaedic Surgery, Amsterdam Movement Sciences, Amsterdam UMC, Academic Medical Centre, University of Amsterdam, Amsterdam, the Netherlands; 5 Department of Radiology, OLVG, Amsterdam, the Netherlands

**Keywords:** Ceramic, migration, polyethylene, radiostereometric analysis, total hip arthroplasty

## Abstract

**Introduction:**

The inelasticity of ceramic bearings might affect primary stability and migration of implants in press-fit total hip arthroplasty (THA). This randomised controlled trial compares migration patterns of the uncemented Delta-TT cup and H-MAX S stem between ceramic and polyethylene liners, up to 5 years follow-up.

**Methods:**

Patients receiving primary press-fit THA were randomly allocated to a ceramic (*n* *=* 28) or polyethylene (*n* *=* 25) liner. Migration was measured using model-based radiostereometric analysis (RSA) at baseline and 1.5, 3, 6, 12, 24 and 60 months postoperatively and compared between groups using mixed models statistical analysis. The focus of this study is on the 2- to 5-year migration of the Delta-TT cup and migration during complete follow-up of the H-MAX S stem up to 5-years.

**Results:**

At 5-year follow-up, mean (95% CI) proximal cup translation was 0.56 (0.37–0.74) mm in ceramic and 0.58 (0.25–0.90) mm in polyethylene (*p* *=* 0.729). The mean change in adduction was 1.05° (0.27–1.82°) in ceramic and 0.78° (−0.16–1.71°) in polyethylene. Mixed modelling showed that all between-group effects were ⩽0.20 mm for translation and ⩽0.22° for rotation at 5 years postoperatively (*p* ⩾ 0.23). Mean cup migration between 2 and 5 years was limited (all parameters <0.17 mm and <0.30°). At 5-year follow-up, mean stem subsidence was 2.09 mm (0.89–3.29 mm) in ceramic and 2.55 (0.97–4.12) mm in polyethylene. The mean change in internal rotation was 3.69° (1.98–5.40°) in ceramic and 4.01° (2.20–5.81°) in polyethylene. Most stem migration occurred up to 1.5 months, stabilising afterwards. All between-group effects were ⩽0.75 mm for translations and ⩽1.41° for rotations (*p* ⩾ 0.26).

**Conclusions:**

5-year migration patterns of press-fit cups and stems were similar between ceramic and polyethylene liners. The Delta-TT cup and H-MAX S stem showed secondary stabilisation and remained stable up to 5 years in both groups, which is promising for long-term survival with both liner types.

**Clinical trial registration:**

ClinicalTrials.gov (NCT03093038)

## Introduction

One of the main reasons for long-term revision in press-fit total hip arthroplasty (THA) is aseptic loosening, often caused by wear induced osteolysis of polyethylene (PE) liners.^1,2^ While highly cross-linked PE (HXLPE) has been developed to decrease wear rates, ceramic-on-ceramic (CoC) shows even lower wear rates.^3^ However, a disadvantage of ceramic (CE) is its higher stiffness compared to polyethylene (PE), while an elasticity modulus similar to bone is known to reduce stress-shielding, promoting osseointegration.^4^ An observational registry study shows a higher risk of 2-year cup revision in CoC compared to CoPE in press-fit THA.^5^ We theorise that CoC could cause a more direct load transfer to the bone-implant interface and increase micromotion of the implant, which could jeopardise osseointegration and the transition to long-term stability.^6^

Initial stability of the implant is an important factor in order for osseointegration to occur. To promote osseointegration, implants are continuously being developed, which has resulted in trabecular titanium implants such as the Delta-TT cup and the hydroxyapatite coated rough macro-textured H-MAX S stem (LimaCorporate, Villanova San Daniele del Friuli, Italy).^7–10^ Stability of the implant can be accurately assessed by measuring migration patterns using radiostereometric analysis (RSA), which can predict the risk for long-term aseptic loosening.^11–13^ Previously published 2-year results of this study showed a trend of more cup migration on some parameters in the CoC group compared to the CoPE group; however, differences were small and not statistically significant.^14^ Results with a longer follow-up on stability of press-fit cups and stems between CoC and CoPE by means of RSA will contribute to our knowledge of the most optimal bearing choice in THA.

Therefore, the objective of this randomised controlled trial was to compare 5-year migration patterns of both the Delta-TT cup and the H-MAX S stem between either a CE or PE liner. To our knowledge, this is the first study to: (1) compare mid-term results between CoC and CoPE bearings by means of RSA; (2) to assess stability of the uncemented H-MAX S stem; and (3) to assess 5-year RSA results of the Delta-TT cup.

## Methods

### Ethical approval

Ethical approval was granted by the local medical ethics committee (registration number NL44230.100.13) and the study was registered at ClinicalTrials.gov (NCT03093038). The design and reporting were performed in accordance with the Consolidated Standards of Reporting Trials (CONSORT) principles and conducted according to the Declaration of Helsinki.

### Study design

This single-centre randomised controlled trial (RCT) was performed on patients between 18 and 75 years of age undergoing primary unilateral press-fit THA at the OLVG (Amsterdam, the Netherlands) between October 2014 and February 2016. All patients received the Delta-TT cup and H-MAX S stem (LimaCorporate) and ceramic 32-mm head (CeramTec GmbH, Plochingen, Germany). Patients were randomised to either a highly cross-linked ultra-high molecular weight polyethylene liner (UHMWPE X-Lima, LimaCorporate) or a BIOLOX delta ceramic liner (CeramTec GmbH) after providing formal written informed consent. Patient demographics, medical history, postoperative complications and patient-reported outcome measures (PROMs) were collected. PROMs up to 5-years postoperatively measured quality of life using the EuroQol 5-dimensions (EQ5D-3L) and physical function using the Hip disability and Osteoarthritis Outcome Score Physical Function Short form (HOOS-PS) and Oxford Hip Score (OHS). Since results on migration of the cup up to 2 years have been previously published, this study focuses on the 2–5-year results of the Delta-TT cup and the complete 5-year migration results of the H-MAX S stem. For further details on patient eligibility, informed consent, surgical procedure, implant specification, sample size calculation and RSA set-up, we refer to the previously published results.^14^

### Radiostereometric analysis

The primary outcome was migration measured with RSA of the acetabular and femoral component over time. Baseline RSA radiographs were acquired within 3 days postoperative before weight-bearing and follow-up radiographs at 1.5, 3, 6, 12, 24 and 60 months after implantation. Double examinations of RSA images were performed at 1-year follow-up to measure precision of the RSA technique (Table 1). The anonymised RSA radiographs were analysed using model-based RSA Software, version 4.2 (RSA*core*, Department of Orthopaedics, LUMC, the Netherlands). Cup migration was calculated using a 3D Hemispherical Elementary Geometrical Shape (EGS) model.^15^ Stem migration was calculated using a combined 3D stem and femoral head model, based on computer aided design (CAD) information.^16^ Migration was calculated following the recommendations of Valstar et al.^17^ The meaning of positive translation (X-axis: medialisation, Y-axis: cranialisation, Z-axis: anterior migration) and positive rotation (X-axis: anterior tilt, Y-axis: internal rotation or anteversion, Z-axis: adduction/decrease of inclination). For the stem, maximum total point motion (MTPM) was calculated, which is the translation of the point on the stem model that moved the most. To prevent loss of data, a marker configuration model (MC-model) was used when necessary.^18^

**Table 1.** Precision calculation presented as the upper limit of the 95% confidence interval (mean + (1.96*SD)) of the measurement error of the double RSA examination at 1-year follow-up.

**Implant**	**Translation (mm)**			**Rotation (degrees)**			**MTPM (mm)**
	*Lateral-medial (X)*	*Distal-proximal (Y)*	*Posterior-anterior (Z)*	*Anterior tilt (X)*	*Internal rotation (Y)*	*Adduction (Z)*	
Delta-TT cup (*n* ***=*** **49)**	0.441	0.213	0.368	0.734	0.955	0.686	-
	*Lateral-medial (X)*	*Distal-proximal (Y)*	*Posterior-anterior (Z)*	*Anterior tilt (X)*	*Internal rotation (Y)*	*Adduction (Z)*	
H-MAX S stem (*n* ***=*** **46)**	0.185	0.463	0.328	0.293	1.034	0.243	1.101

MTPM, maximum total point motion.

### Explorative analysis

As an explorative analysis, the relation between cup and stem total translation (TT) was investigated using Pearson’s Correlation coefficient. The aim was to investigate whether increased migration in one component is correlated with increased migration in the other component. We hypothesised that micromotion in the cup and stem could be related, especially with a stiff bearing. TT was calculated to approximate overall translation of the implant and was defined as: √((translation X-axis)^2^ + (translation Y-axis)^2^ + (translation Z-axis)^2^) for both the cup and stem.^11,19,20^ Outcomes were presented in scatter plots during the settling phase at 1.5, 3 and 6 months and in a table for all time points.

### Statistics

The initial power analysis was performed for the primary outcome (cup migration at 2 years). We aimed for a minimum of 16 patients in each group at 5-year follow-up, to be able to detect a difference between groups with the magnitude of 1 standard deviation, with 80% power and alpha = 0.05. Statistical analyses were performed with SPSS Statistics version 27.0 (IBM Corp. Armonk, New York, USA). To assess cup and stem migration a mixed model analysis was performed, with bearing (PE vs. CE) as the primary independent value of interest. Primary outcome was the effect of the bearing over the 5-year follow-up period on implant migration. Group differences were separately analysed at each time point including time as a categorical factor variable and a time-by-group interaction term. Differences were considered significant for *p*-values below 0.05.

## Results

**Figure 1.**
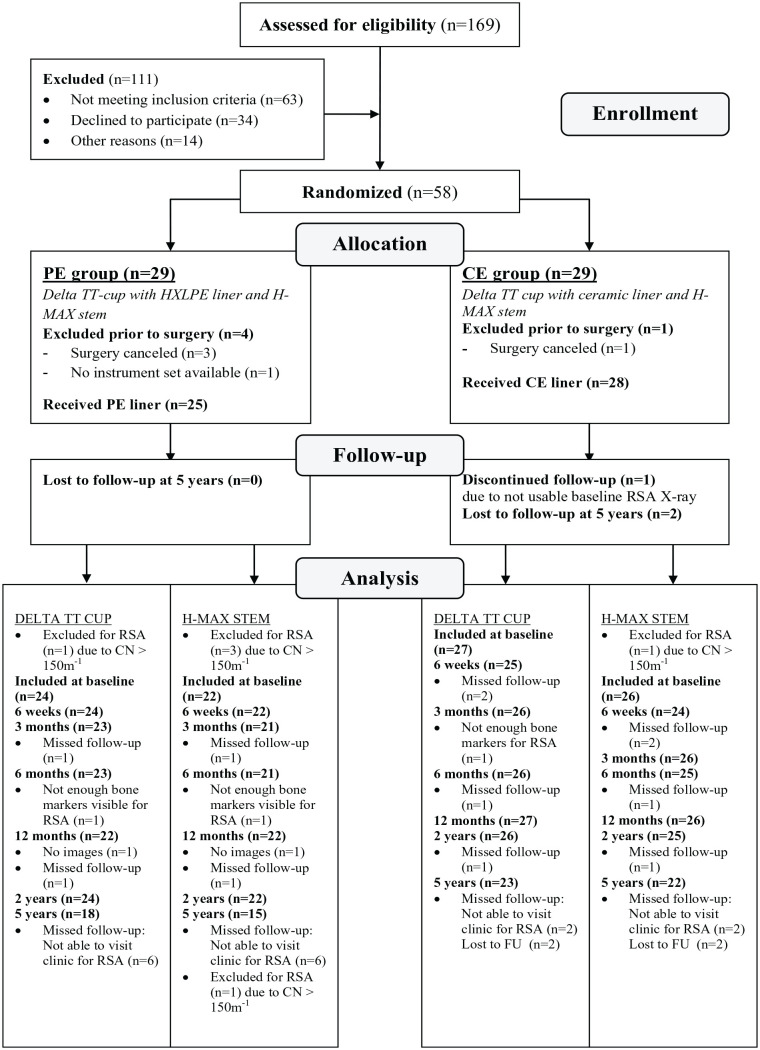
Flowchart of patient selection.

**Figure 2.**
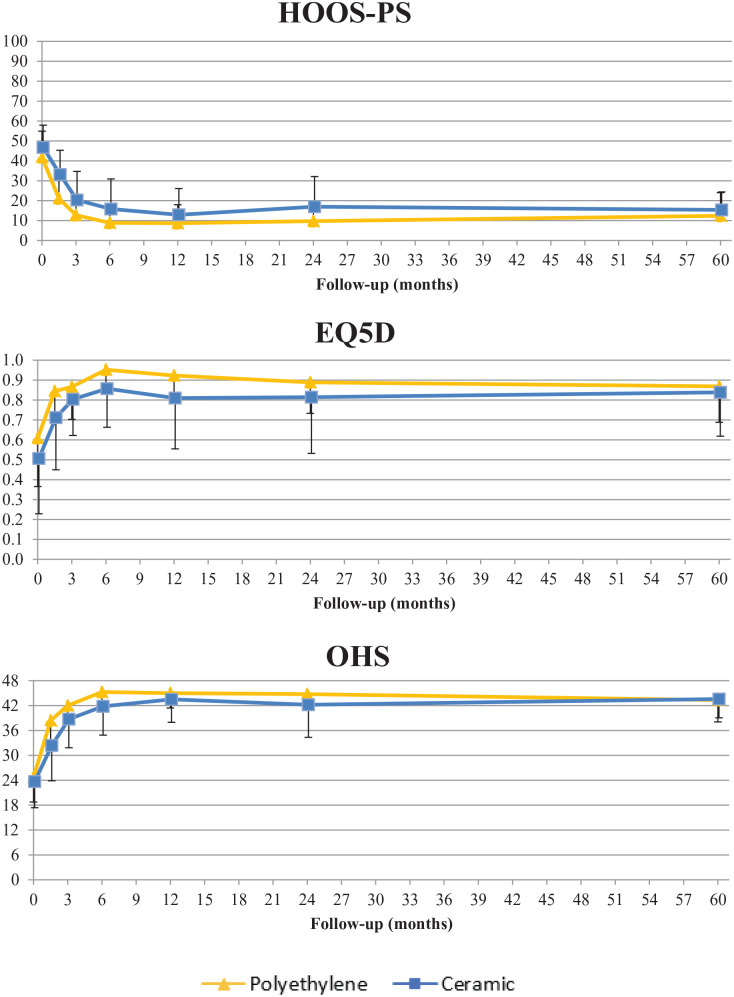
Mean (SD) improvement in patient-reported outcomes (PROMs) in the 2 study groups throughout the 5-year follow-up period.

A total of 28 patients were included in the CE group and 25 patients in the PE group. Figure 1 presents the flowchart of patient selection and follow-up. Demographic details and implant information are reported in Table 2. As is shown in Figure 2, improvement in patient-reported outcomes over time was similar in the 2 groups and clinically relevant from baseline up to 5 years. An MC-model was used for 2 cups (1 CE, 1 PE) and 2 stems (1 CE, 1 PE) to prevent data loss. At 5-year follow-up, 23 Delta-TT cups and 22 H-MAX S stems were available in the CE group and 18 Delta-TT cups and 15 H-MAX S stems were available in the PE group for RSA analysis (Figure 1).

**Table 2.** Demographic details, indication for THA and implant information for the 2 study groups. Age and BMI are reported as mean (range) and all other values as number (%).

**Variable**	**CE GROUP** *n* = 27	**PE GROUP** *n* = 25
**Age (years)**	58.2 (40.0 – 71.0)	59.8 (40.0 – 70.0)
**BMI (kg/m^2^)**	25.7 (19.8 – 35.4)	27.4 (20.0 – 35.4)
**Sex (male)**	14 (52)	14 (56)
**Indication for THA**		
**-** Primary OA	24 (88.9)	24 (96)
**-** OA and DDH	2 (7.4)	1 (4)
**-** Osteonecrosis	1 (3.7)	-
**Cup size**		
- 50	3 (11.1)	5 (20)
- 52	4 (14.8)	2 (8)
- 54	4 (14.8)	7 (28)
- 56	11 (40.7)	3 (12)
- 58	2 (7.4)	3 (12)
- 60	3 (11.1)	4 (16)
- 62	-	1 (4)
**Stem size**		
- 8	1 (3.7)	-
- 9	5 (18.5)	3 (12)
- 10	6 (22.2)	3 (12)
- 11	3 (11.1)	6 (24)
- 12	6 (22.2)	8 (32)
- 13	3 (11.1)	4 (16)
- 14	2 (7.4)	-
- 15	1 (3.7)	-
- 16	-	1 (4)

CE, ceramic; PE, polyethylene; CI, confidence interval; THA, total hip arthroplasty; BMI, body mass index; OA, osteoarthritis; DDH, developmental dysplasia of the hip; PE, polyethylene; CE, ceramic.

### Radiostereometric analysis of the cup

**Figure 3.**
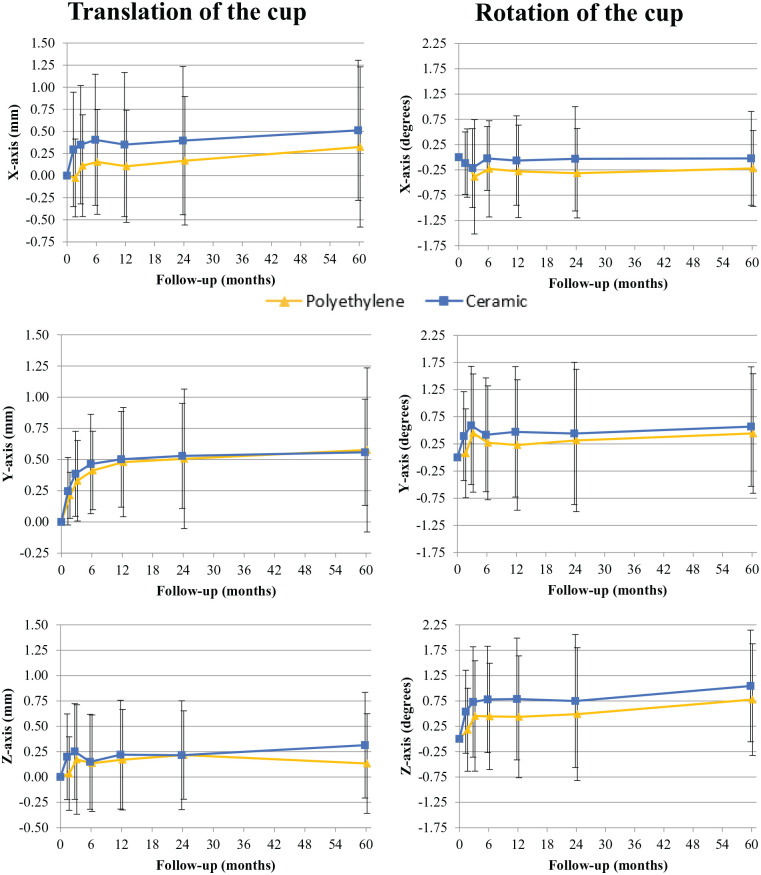
Mean (SD) translation and rotation of the Delta TT cup with a polyethylene (PE) or ceramic (CE) liner over time.

Mean migration of the cup with a CE or PE liner is displayed in Figure 3 and presented in detail with between group effects up until 5-year follow-up in Table 3. Between group effects between CE and PE were not different at any time point (all *p* *>* 0.108). Limited migration of the Delta-TT cup was observed between 2- and 5-year follow-up: mean (and 95% confidence interval [CI]) proximal translation of 0.53 mm (0.36–0.70 mm) and 0.56 mm (0.37–0.74 mm) in the CE group and 0.51 mm (0.27–0.74 mm) and 0.58 mm (0.25–0.90 mm) in the PE group at 2 and 5 years respectively. For inclination (Z-axis) a mean adduction of 0.75*°* (0.01–1.49*°*) and 1.05*°* (0.27–1.82*°*) in the CE group and 0.49*°* (−0.18–1.16*°*) and 0.78*°* (−0.16–1.71*°*) in the PE group was observed at 2 and 5 years respectively.

**Table 3.** Mean migration of the cup in all directions at all time points during follow-up.

Outcome		CE				PE				CE vs. PE			
				95% CI				95% CI			95% CI		
		*n*	Mean	Lower bound	Upper bound	*n*	Mean	Lower bound	Upper bound	Between group diff.	Lower bound	Upper bound	*p*-value
**Tx**	1.5 mo	25	0.30	0.03	0.56	24	−0.03	−0.21	0.16	0.31	−0.07	0.70	0.108
	3 mo	26	0.35	0.08	0.62	23	0.11	−0.14	0.36	0.21	−0.18	0.60	0.279
	6 mo	26	0.41	0.11	0.70	23	0.16	−0.10	0.41	0.25	−0.14	0.63	0.204
	1 yr	27	0.35	0.03	0.67	22	0.11	−0.18	0.39	0.26	−0.13	0.64	0.188
	2 yr	26	0.40	0.06	0.73	24	0.17	−0.14	0.48	0.23	−0.15	0.62	0.231
	5 yr	23	0.51	0.17	0.86	18	0.32	−0.13	0.77	0.20	−0.19	0.59	0.315
**Ty**	1.5 mo	25	0.25	0.13	0.36	24	0.21	0.14	0.29	0.03	−0.19	0.25	0.769
	3 mo	26	0.39	0.25	0.52	23	0.33	0.19	0.47	0.06	−0.16	0.27	0.613
	6 mo	26	0.46	0.30	0.63	23	0.41	0.28	0.55	0.05	−0.16	0.27	0.620
	1 yr	27	0.50	0.35	0.65	22	0.48	0.29	0.67	0.03	−0.19	0.25	0.767
	2 yr	26	0.53	0.36	0.70	24	0.51	0.27	0.74	0.02	−0.20	0.23	0.889
	5 yr	23	0.56	0.37	0.74	18	0.58	0.25	0.90	−0.04	−0.26	0.18	0.729
**Tz**	1.5 mo	25	0.20	0.03	0.37	24	0.03	−0.12	0.19	0.13	−0.13	0.29	0.321
	3 mo	26	0.25	0.06	0.44	23	0.17	−0.06	0.41	0.06	−0.20	0.33	0.626
	6 mo	26	0.15	−0.04	0.34	23	0.14	−0.07	0.34	0.01	−0.25	0.27	0.948
	1 yr	27	0.22	0.01	0.43	22	0.17	−0.05	0.39	0.05	−0.21	0.31	0.697
	2 yr	27	0.22	−0.001	0.43	24	0.22	0.03	0.40	−0.02	−0.28	0.24	0.893
	5 yr	23	0.31	0.09	0.54	18	0.13	−0.11	0.38	0.16	−0.10	0.43	0.227
**Rx**	1.5 mo	25	−0.12	−0.37	0.14	24	−0.11	−0.40	0.17	0.02	−0.45	0.50	0.917
	3 mo	26	−0.21	−0.53	0.10	23	−0.39	−0.88	0.10	0.19	−0.28	0.66	0.426
	6 mo	26	−0.02	−0.28	0.23	23	−0.23	−0.64	0.18	0.13	−0.34	0.60	0.578
	1 yr	27	−0.06	−0.41	0.28	22	−0.28	−0.68	0.13	0.20	−0.28	0.67	0.409
	2 yr	26	−0.03	−0.45	0.39	24	−0.31	−0.69	0.06	0.29	−0.18	0.76	0.219
	5 yr	23	−0.02	−0.42	0.38	18	−0.22	−0.59	0.15	0.17	−0.30	0.65	0.471
**Ry**	1.5 mo	25	0.39	−0.07	0.86	24	0.08	−0.27	0.42	0.27	−0.41	0.96	0.430
	3 mo	26	0.59	0.02	1.16	23	0.45	−0.02	0.92	0.11	−0.58	0.79	0.753
	6 mo	26	0.42	−0.10	0.94	23	0.27	−0.18	0.72	0.25	−0.43	0.94	0.460
	1 yr	27	0.47	−0.11	1.05	22	0.23	−0.30	0.76	0.25	−0.44	0.93	0.473
	2 yr	26	0.44	−0.16	1.05	24	0.31	−0.24	0.87	0.11	−0.57	0.79	0.750
	5 yr	23	0.57	−0.08	1.21	18	0.44	−0.11	0.99	0.11	−0.59	0.80	0.759
**Rz**	1.5 mo	25	0.54	0.04	1.03	24	0.18	−0.19	0.55	0.36	−0.47	1.18	0.389
	3 mo	26	0.73	0.14	1.32	23	0.45	−0.08	0.99	0.26	−0.56	1.08	0.525
	6 mo	26	0.78	0.12	1.44	23	0.45	−0.13	1.03	0.36	−0.46	1.18	0.389
	1 yr	27	0.79	0.11	1.47	22	0.44	−0.18	1.05	0.40	−0.42	1.22	0.337
	2 yr	26	0.75	0.01	1.49	24	0.49	−0.18	1.16	0.27	−0.55	1.09	0.516
	5 yr	23	1.05	0.27	1.82	18	0.78	−0.16	1.71	0.22	−0.62	1.06	0.607

CE, ceramic; PE, polyethylene; CI, confidence interval.

For translation, between group effects over a 5-year period on the X, Y and Z-axis were 0.25 mm (95% CI, −0.12–0.62 mm; *p* *=* 0.181), 0.03 mm (95% CI, −0.17–0.24 mm; *p* *=* 0.736) and 0.06 mm (95% CI −0.18–0.31 mm; *p* *=* 0.589). For rotation, between group effects over a 5-year period on the X, Y and Z-axis were respectively 0.17*°* (95% CI −0.27–0.61*°; p* *=* 0.452), 0.19*°* (95% CI, −0.47–0.85*°; p* *=* 0.562) and 0.37*°* (95% CI −0.40–1.14*° p* *=* 0.337). In some patients, fewer bone markers were available for analysis at 5-year follow-up and therefore migration calculations for the entire follow-up were performed with a reduced number of available markers.^17^ Therefore, results of cup migration up to 2 years postoperatively in this study may differ slightly from the previously published results. Individual cup migration patterns are presented in Appendices 1 and 2. 1 cup with a PE insert, showed increased and ongoing migration on all parameters, apart from Y-axis rotation, up to 5 years postoperatively. No potential causes were found for ongoing cup migration in this patient, nor was this patient at risk for low bone quality (male, high body mass index [BMI] and age <50 years at the time of inclusion). Mid-term, the increase in cranial migration reduced, with 2.0 mm at 1 year, 2.8 mm at 2 years and 3.0 mm at 5 years postoperatively. Separate analysis without this cup showed no significant outcomes.

### Radiostereometric analysis of the stem

**Figure 4.**
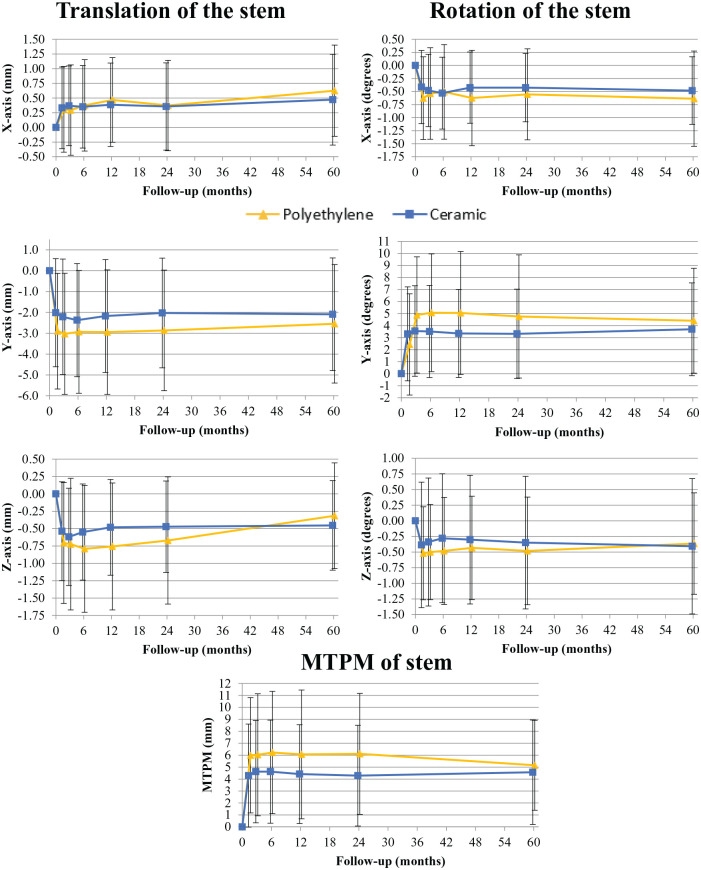
Mean (SD) translation, rotation and MTPM of the H-MAX S stem with a polyethylene (PE) or ceramic (CE) liner over time.

Migration of the stem with a CE or PE liner is shown in Figure 4 and presented in detail with between group effects up to 5-year follow-up in Table 4. Between group effects for mean migration were not different between CE and PE at any time point (*p* *>* 0.173). Migration of the stem occurred mostly in the first 1.5 months postoperatively and remained stable after this time point. Stem migration was most pronounced for negative translation on the Y-axis (i.e. subsidence), rotation on the Y-axis and MTPM. At 5-year follow-up, H-MAX S stems showed mean subsidence in the CE group of 2.09 mm (0.89–3.29 mm) and 2.55 mm (0.97–4.12 mm) in the PE group.

**Table 4.** Migration of the stem in all directions at all time points during follow-up.

Outcome		CE				PE				CE vs. PE			
				95% CI				95% CI			95% CI		
		*n*	Mean	Lower bound	Upper bound	*n*	Mean	Lower bound	Upper bound	Between group diff.	Lower bound	Upper bound	*p*-value
**Tx**	1.5 mo	24	0.33	0.04	0.63	22	0.31	−0.01	0.63	0.04	−0.38	0.46	0.857
	3 mo	26	0.37	0.09	0.64	21	0.29	−0.06	0.65	0.07	−0.35	0.49	0.750
	6 mo	25	0.35	0.06	0.64	21	0.38	0.02	0.73	−0.05	−0.47	0.37	0.818
	1 yr	26	0.39	0.10	0.67	20	0.47	0.13	0.80	−0.00	−0.42	0.42	0.985
	2 yr	25	0.36	0.05	0.66	22	0.37	0.03	0.72	0.01	−0.41	0.43	0.950
	5 yr	22	0.47	0.13	0.82	15	0.63	0.20	1.06	−0.03	−0.48	0.39	0.899
**Ty**	1.5 mo	24	−2.01	−3.11	−0.92	22	−2.90	−4.13	−1.67	0.67	−0.91	2.24	0.401
	3 mo	26	−2.21	−3.33	−1.09	21	−3.03	−4.35	1.70	0.72	−0.86	2.30	0.362
	6 mo	25	−2.37	−3.49	−1.25	21	−2.93	−4.27	−1.59	0.60	−0.97	2.18	0.447
	1 yr	26	−2.17	−3.27	−1.08	20	−2.95	−4.35	−1.55	0.68	−0.90	2.26	0.388
	2 yr	25	−2.03	−3.11	−0.94	22	−2.87	−4.15	−1.58	0.68	−0.89	2.26	0.388
	5 yr	22	−2.09	−3.29	−0.89	15	−2.55	−4.12	−0.97	0.75	−0.83	2.33	0.345
**Tz**	1.5 mo	24	−0.54	−0.84	−0.24	22	−0.71	−1.09	−0.32	0.13	−0.33	0.59	0.572
	3 mo	26	−0.62	−0.90	−0.34	21	−0.72	−1.15	−0.29	0.11	−0.34	0.57	0.621
	6 mo	25	−0.55	−0.84	−0.26	21	−0.79	−1.21	−0.38	0.23	−0.23	0.69	0.316
	1 yr	26	−0.48	−0.76	−0.20	20	−0.76	−1.18	−0.33	0.26	−0.20	0.71	0.264
	2 yr	25	−0.47	−0.74	−0.20	22	−0.67	−1.08	−0.26	0.15	−0.30	0.61	0.507
	5 yr	22	−0.45	−0.74	−0.17	15	−0.31	−0.74	0.11	−0.02	−0.48	0.44	0.942
**Rx**	1.5 mo	24	−0.41	−0.71	−0.12	22	−0.63	−0.98	−0.28	0.18	−0.26	0.63	0.412
	3 mo	26	−0.48	−0.76	−0.20	21	−0.54	−0.94	−0.14	0.03	−0.41	0.47	0.908
	6 mo	25	−0.53	−0.81	−0.25	21	−0.51	−0.92	−0.09	0.02	−0.43	0.46	0.940
	1 yr	26	−0.43	−0.70	−0.15	20	-0.62	−1.05	−0.20	0.15	−0.30	0.59	0.506
	2 yr	25	−0.43	−0.70	−0.16	22	−0.55	−0.94	−0.17	0.08	−0.36	0.53	0.710
	5 yr	22	−0.48	−0.77	−0.19	15	−0.64	−1.14	−0.13	0.08	−0.37	0.53	0.712
**Ry**	1.5 mo	24	3.31	1.66	4.96	22	4.87	2.71	7.04	−1.48	−3.96	1.00	0.235
	3 mo	26	3.55	2.03	5.07	21	4.90	2.67	7.12	−1.42	−3.91	1.05	0.254
	6 mo	25	3.51	1.93	5.09	21	5.24	2.96	7.52	−1.71	−4.19	0.77	0.173
	1 yr	26	3.35	1.87	4.82	20	4.85	2.34	7.35	−1.57	−4.05	0.91	0.210
	2 yr	25	3.32	1.78	4.85	22	5.11	2.84	7.37	−1.66	−4.13	0.822	0.185
	5 yr	22	3.69	1.98	5.40	15	4.01	2.20	5.81	−1.41	−3.90	1.07	0.260
**Rz**	1.5 mo	24	−0.39	−0.81	0.04	22	−0.52	−0.85	−0.19	0.18	−0.35	0.71	0.509
	3 mo	26	−0.34	−0.75	0.07	21	−0.50	−0.85	−0.15	0.19	−0.34	0.72	0.470
	6 mo	25	−0.28	−0.70	0.15	21	−0.48	−0.87	−0.09	0.19	−0.34	0.72	0.474
	1 yr	26	−0.30	−0.71	−0.12	20	−0.30	−0.72	0.12	0.13	−0.40	0.66	0.618
	2 yr	25	−0.35	−0.79	0.09	22	−0.48	−0.86	−0.10	0.14	−0.39	0.67	0.609
	5 yr	22	−0.41	−0.89	0.07	15	−0.36	−0.81	0.08	0.07	−0.47	0.60	0.807
**M** **T** **P** **M**	1.5 mo	24	4.29	2.47	6.11	22	5.99	3.85	8.13	-1.52	−4.15	1.10	0.249
	3 mo	26	4.63	2.90	6.35	21	6.03	3.71	8.35	−1.39	−4.01	1.24	0.294
	6 mo	25	4.62	2.84	6.40	21	6.22	3.89	8.55	−1.58	−4.21	1.04	0.232
	1 yr	26	4.42	2.75	6.09	20	6.07	3.55	8.59	−1.55	−4.18	1.08	0.241
	2 yr	25	4.29	2.55	6.03	22	6.11	3.87	8.35	−1.63	−4.26	0.99	0.218
	5 yr	22	4.57	2.64	6.51	15	5.16	3.08	7.24	−1.48	−4.11	1.15	0.264

CE, ceramic; PE, polyethylene; CI, confidence interval.

For translation, between group effects over a 5 year period on the X, Y and Z-axis were 0.01 mm (95% CI, −0.41–0.43 mm; *p* *=* 0.952), 0.68 mm (95% CI −0.89–2.26 mm; *p* *=* 0.388) and 0.15 mm (95% CI, −0.29–0.60 mm; *p* *=* 0.492). For rotation, between group effects on the X, Y and Z-axis were 0.09*°* (95% CI, −0.34–0.52*°; p* *=* 0.677), −1.54*°* (95% CI, −4.00–0.93*°; p* *=* 0.215) and 0.15*°* (95% CI, −0.37–0.68*°; p* *=* 0.564) respectively. MTPM showed a between group effect of −1.52 mm (95% CI, −4.14–1.10 mm, *p* *=* 0.249). Individual stem migration patterns are presented in Appendices 3 and 4.

### Explorative analysis cup versus stem migration

**Figure 5.**
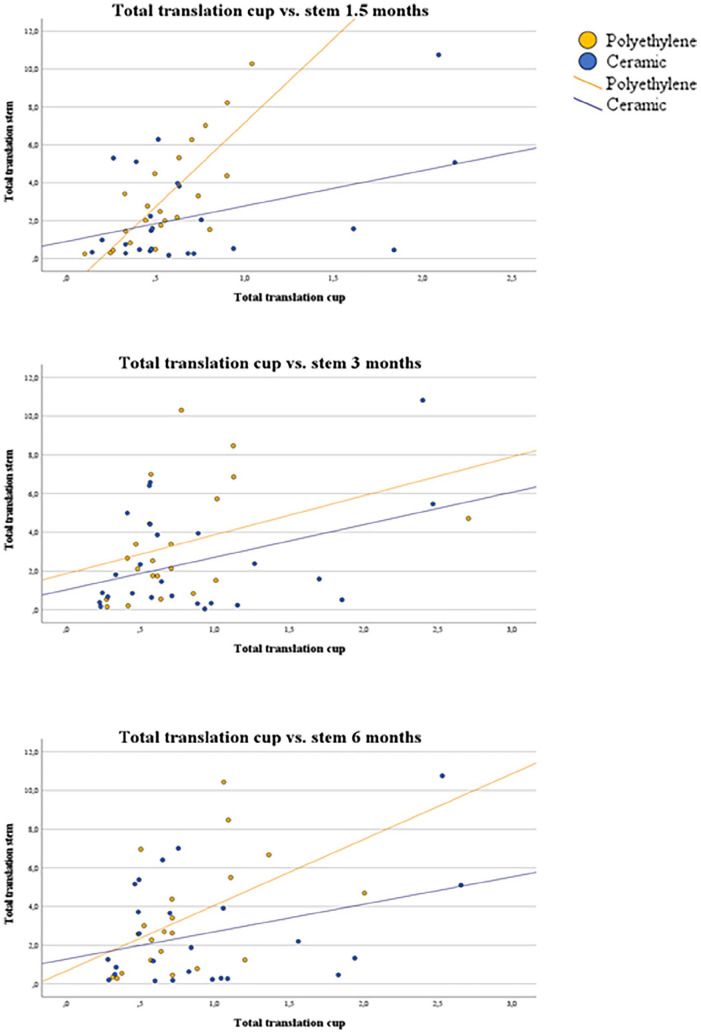
Correlation between total translation of the cup versus the stem.

All correlation coefficients for total translation were positive, indicating that more migration of the cup coincided with more migration of the stem (Table 5) (Figure 5). Pearson correlation coefficients ranged from 0.23 (12 months) to 0.41 (1.5 months) in the CE group and from 0.31 (60 months) to 0.78 (1.5 months) in the PE group.

**Table 5.** Pearson correlation coefficients (r) for total translation of the cup versus the stem.

	Time (months)	Ceramic				Polyethylene			
		r	95% CI		*p*-value	r	95% CI		*p*-value
			Lower bound	Upper bound			Lower bound	Upper bound	
**Total translation**	**1.5**	0.41	0.01	0.70	0.05	0.78	0.53	0.90	0.00
	**3**	0.39	−0.00	0.68	0.05	0.36	−0.09	0.69	0.11
	**6**	0.34	−0.06	0.65	0.09	0.48	0.06	0.76	0.03
	**12**	0.23	−0.17	0.57	0.25	0.34	−0.13	0.68	0.15
	**24**	0.32	−0.09	0.63	0.13	0.36	−0.08	0.68	0.10
	**60**	0.36	−0.08	0.68	0.10	0.31	−0.24	0.71	0.26

CI, confidence interval.

### Complications

1 patient (CE group) needed early revision of the femoral head and antibiotic treatment due to a periprosthetic joint infection. Another patient (PE group) visited the hospital 3 times because of recurrent dislocation and pain in the groin and a distally migrated stem. This stem showed subsidence of 8 mm at 1.5 months postoperatively which stabilised afterwards and Y-axis rotation was 7° and 10° at 1.5 and 24 months respectively. In retrospect, a larger stem size might have been a better fit for this patient. Revision surgery of the stem and liner was performed at 3.5 years postoperatively. At 5 years postoperatively there were no clinical signs of aseptic loosening for any of the cups or any of the remaining stems. 2 patients reported occasional squeaking in the operated hip with a CoC bearing; however, they did not require revision surgery.

## Discussion

This randomised controlled RSA trial with 5-year follow-up found no significant differences in mean migration of press-fit cups and stems between CoC and CoPE bearings. While a trend is seen of more migration on some parameters for Delta-TT cups in the CE group and for H-MAX S stems in the PE group, between-group effects are small and none reach statistical significance. To our knowledge only 1 other RCT RSA study, besides the previously published 2-year results of this study, has assessed cup migration between CoC and CoPE in press-fit THA, both showing no difference at 2 years postoperatively.^14,20^ Perticarini et al.^21^ also showed safe ingrowth of the Delta-TT cup without using RSA at 5 years.

Important parameters predicting aseptic loosening of the cup are cranial migration and change in inclination.^11,12,22^ The threshold proposed by Pijls et al.^12^ considers mean proximal cup translation <0.2 mm as acceptable and >1.0 mm at 2 years as unacceptable. According to these thresholds, the Delta-TT cup would be classified ‘at risk’ (between 0.2 mm and 1.0 mm) for having revision rates >5% at 10 years. However, the majority of cups in Pijls et al.^12^ were classified ‘at risk’ while showing long-term revision rates <5%. Moreover, only a limited number of press-fit RSA studies were included. Press-fit components are expected to show more migration compared to cemented components because of the settling phase; therefore, more research is needed to develop specific migration thresholds for press-fit cups. Nieuwenhuijse et al.^11^ proposed thresholds for individual patient proximal cup translation of 1.76 mm and abduction of 2.53*°* about the Z-axis at 2 years. In our study only 1 cup in the PE group showed mid-term migration above the threshold for proximal translation, indicating that this cup might be at risk for long-term aseptic loosening.

A recent registry study in the Dutch Arthroplasty Register (LROI) showed a higher 2-year cup revision rate in press-fit THA in CoC compared to CoPE, with cup loosening as the only significant different reason for revision.^5^ The registry study included a higher number of patients than our study and analysed the 3 most frequently used cups available. While a larger sample size increases the likelihood of detecting significant differences, registry studies cannot conclude on causality. In our study patients were randomised to minimise the risk of bias and all patients received the same cup, stem and ceramic head.

The literature shows that both low bone mineral density (BMD) and impaired bone quality caused by rheumatoid arthritis (compared to osteoarthritis) contribute to increased cup migration.^23,24^ More research is needed to investigate whether the use of a stiff CE liner in these specific subgroups compromises osseointegration.

Focusing on the stem, most migration was seen in the first 1.5 months after surgery and was mostly subsidence on and rotation about the Y-axis. Slightly higher mean stem migration on all parameters was observed in PE compared to CE, but it did not reach statistical significance. The literature shows that the cup remains the weakest link in THA and is spherical, making it more vulnerable to migration in different directions, whereas conical stem migration would be limited by the surrounding shape of the femur.^25^ Streit et al.^19^ found an association between early subsidence in press-fit collarless stems and aseptic femoral loosening and set a threshold of 2.7mm subsidence at 2-years for individual patients. In our study, 8 out of 25 (32%) H-MAX S stems in the CE group and 9 out of 22 (41%) in PE showed subsidence above this threshold at 2 years. Previous studies mention that different design features, like collarless press-fit stems such as the H-MAX show more early migration, but should not be interpreted as inferior osseointegration.^25,26^ In the literature it is suggested that in press-fit stems, an implant specific approach might be preferred and stabilisation of migration might be more suitable to predict unsafe stems as compared to the absolute value of migration.^19,26^ Therefore, caution is needed when predicting long-term results for stems based on their initial subsidence, especially if this is followed by early stabilisation, as seen in our study and in the literature, which could be part of the normal settling of a prosthesis.^27^

In our study, 1 CE patient required revision surgery of the stem due to recurrent dislocation, which might have been caused by undersizing. This stem showed distal migration of 8 mm at 1.5 months postoperatively. As mentioned above, more H-MAX S stems showed high early subsidence: 2 other patients (1 CE, 1 PE) showed distal migration of >10 mm at 1.5 months postoperatively, rotation about the Y-axis was 4.1° and 4.3° in the first patient and 12.7° and 13.3° in the second patient at 1.5 and 24 months respectively. These subjects were free of complaints during further follow-up and since all stems showed early stabilisation. Considering that only 1 subject required revision, the H-MAX S stem is found to be a safe option for use in THA regardless of bearing type.

Our explorative analysis revealed a positive correlation between cup and stem migration in press-fit THA up to 5 years, in both liner types. To our knowledge, this is the first study to explore this relationship. We theorise that when increased micromotion in the cup and stem are related, osseointegration might be insufficient, potentially due to impaired bone quality. Further research including BMD measurements is needed to investigate this potential correlation and the influence of bearing material.

### Strengths and limitations

An important strength of this study is the randomised RSA design comparing mid-term migration of a press-fit cup and stem between CoC and CoPE bearing. This study is clinically relevant, since RSA is important for the introduction of new implants according to the Dutch Guidelines for THA.^28^ In our study, a variation in individual migration patterns was observed. Variation in migration is known to be higher in press-fit implants compared to cemented implants and may complicate detection of differences in small groups. A limitation of our study is that at 5-year follow-up only 18 cups and 15 stems were available for RSA analysis in the PE group, since some patients were unwilling or not able to visit the clinic due to chronic illness, work or moving away from the hospital. Furthermore, we did not perform BMD measurements. To investigate the influence of bearing on migration more comprehensively, we recommend experiments in larger trials with long-term follow-up. Moreover, we recommend follow-up on long-term clinical functioning of these implants and the incidence of osteolysis, loosening and wear.

In conclusion, observed 5-year migration patterns of press-fit components were similar between ceramic and polyethylene liners. In both groups, the Delta-TT cup and H-MAX S stem showed secondary stabilisation after a phase of initial migration and remained stable up to 5 years. These results are promising for long-term survival with both polyethylene and ceramic liners.

## Figures and Tables

**Appendix 1.**
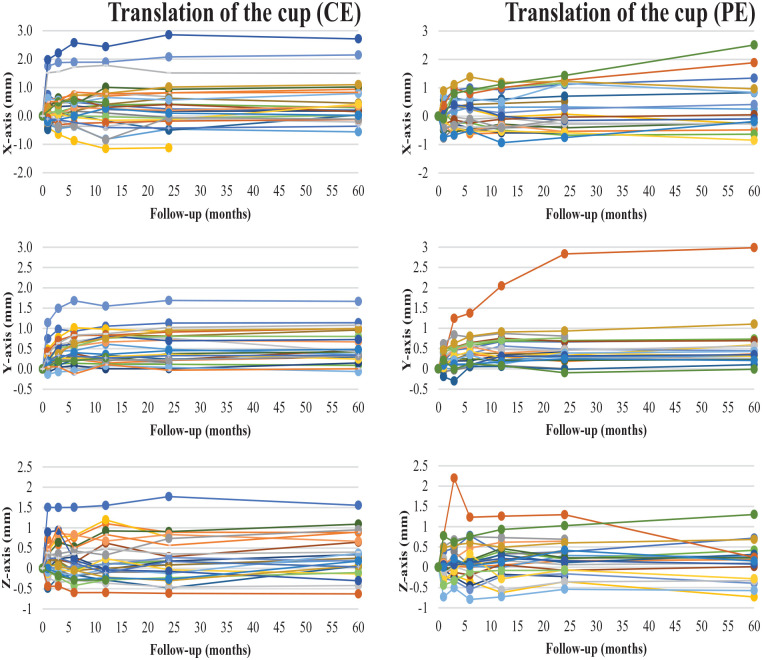
Individual translation patterns of the cup with a ceramic liner (left) or polyethylene liner (right).

**Appendix 2.**
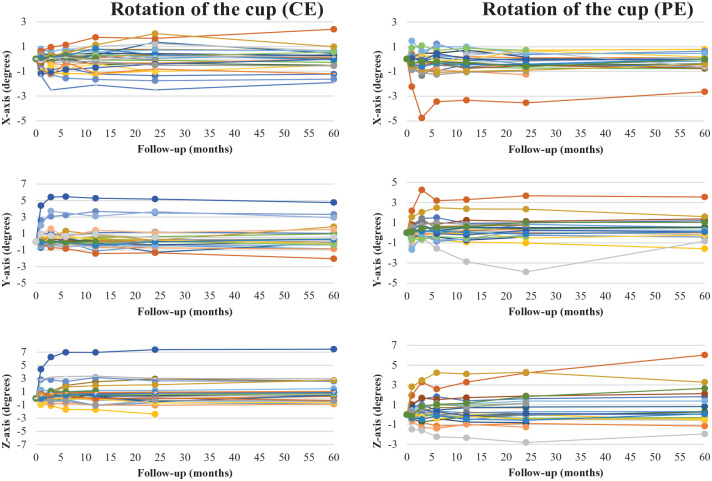
Individual rotation patterns of the cup with a ceramic liner (left) or polyethylene liner (right).

**Appendix 3.**
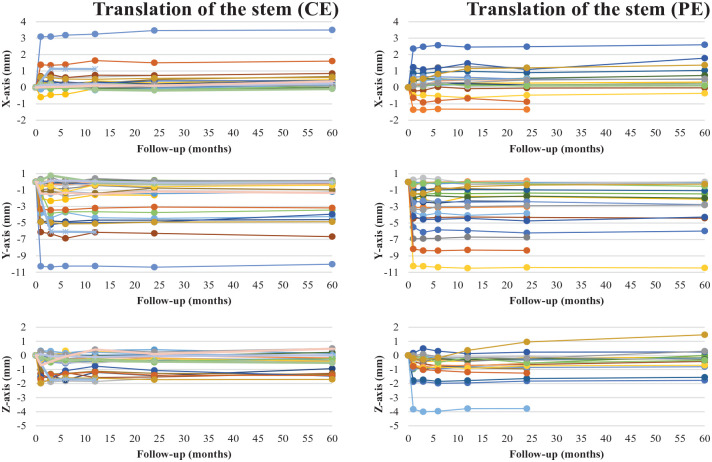
Individual translation patterns of the stem with a ceramic liner (left) or polyethylene liner (right).

**Appendix 4.**
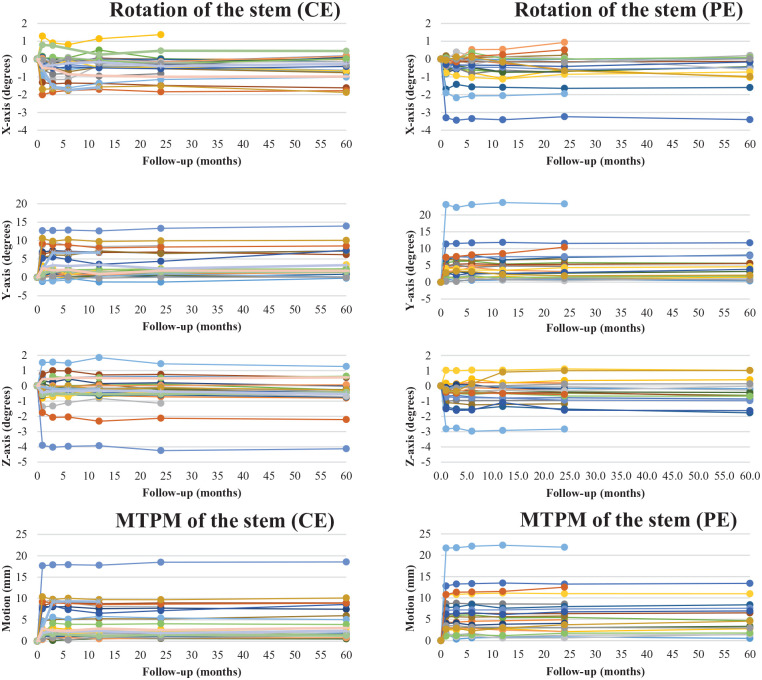
Individual rotation patterns and pattern of Maximum Total Point Motion (MTPM) of the stem with a ceramic liner (left) or polyethylene liner (right).
